# Genotoxic mixtures and dissimilar action: concepts for prediction and assessment

**DOI:** 10.1007/s00204-013-1170-x

**Published:** 2013-12-03

**Authors:** Sibylle Ermler, Martin Scholze, Andreas Kortenkamp

**Affiliations:** Institute for the Environment, Brunel University, Kingston Lane, Uxbridge, Middlesex, UB8 3PH UK

**Keywords:** Aneugen, Clastogen, Genotoxic mixtures, CBMN assay, CHO-K1 cells, Dissimilar action, Similar action

## Abstract

Combinations of genotoxic agents have frequently been assessed without clear assumptions regarding their expected (additive) mixture effects, often leading to claims of synergisms that might in fact be compatible with additivity. We have shown earlier that the combined effects of chemicals, which induce micronuclei (MN) in the cytokinesis-block micronucleus assay in Chinese hamster ovary-K1 cells by a similar mechanism, were additive according to the concept of concentration addition (CA). Here, we extended these studies and investigated for the first time whether valid additivity expectations can be formulated for MN-inducing chemicals that operate through a variety of mechanisms, including aneugens and clastogens (DNA cross-linkers, topoisomerase II inhibitors, minor groove binders). We expected that their effects should follow the additivity principles of independent action (IA). With two mixtures, one composed of various aneugens (colchicine, flubendazole, vinblastine sulphate, griseofulvin, paclitaxel), and another composed of aneugens and clastogens (flubendazole, doxorubicin, etoposide, melphalan and mitomycin C), we observed mixture effects that fell between the additivity predictions derived from CA and IA. We achieved better agreement between observation and prediction by grouping the chemicals into common assessment groups and using hybrid CA/IA prediction models. The combined effects of four dissimilarly acting compounds (flubendazole, paclitaxel, doxorubicin and melphalan) also fell within CA and IA. Two binary mixtures (flubendazole/paclitaxel and flubendazole/doxorubicin) showed effects in reasonable agreement with IA additivity. Our studies provide a systematic basis for the investigation of mixtures that affect endpoints of relevance to genotoxicity and show that their effects are largely additive.

## Introduction

Research into combination effects of genotoxic chemicals has typically employed concepts and approaches that differ in important ways from those underpinning other areas of mixture toxicology. Frequently, synergisms have been declared wherever the observed combined responses were larger than the simple sum of effects, with the implicit assumption that the summation of effects provides valid additivity expectations (Hecker 1976). Even more widely used is an approach based on comparisons between mixture effects and the effects of single components, without reference to null hypotheses about expected additive effects. Here, synergisms are pronounced when the mixture effect is greater than that of the most potent component (for recent examples, see Bouslimi et al. 2008; Kocaman and Topaktas 2010). Due to its lack of additivity expectations, this approach precludes delineations of additive effects from true synergisms (defined as “greater than additive”), with a high chance of claiming synergisms when the combined effects are in fact additive.

The fallacy of using effect summation for deriving additivity expectations as a point of reference for establishing synergisms has been discussed by Berenbaum (1985). Effect summation produces erroneous additivity expectations when calculations are based on the effects of single mixture components that exhibit nonlinear concentration–response curves. Consequently, more reliable methods for defining additivity have been established in other areas of mixture toxicology [reviewed by Kortenkamp et al. (2009)].

Two concepts have found wide application for the calculation of expected additive effects: dose or concentration addition (CA) and independent action (IA). Both concepts utilise algorithms for translating effect concentrations (CA) or effects (IA) of the individual mixture components into expected combined effects, but are based on different assumptions. CA conceptualises the idea that all components in a mixture behave as if they were dilutions of one another (Loewe and Muischnek 1926). If all mixture constituents act on the same molecular target, it is thought that one chemical can be replaced by an equal fraction of an equi-effective concentration (e.g. an EC_50_) of another, without diminishing the overall combined effect. In contrast, IA assumes that a combination effect can be calculated from the responses of the individual mixture components by following the statistical concept of independent random events (Bliss 1939). In the case of simultaneous exposure to several chemicals, the principles of IA are thought to be met only by substances with strictly dissimilar mechanisms of action. The validity of IA for multi-component mixtures under such conditions has been demonstrated in algae (Faust et al. 2003) and in bacteria (Backhaus et al. 2000), but evidence showing its applicability to responses of mammalian cells or whole organisms is missing altogether.

The application of CA and IA, based on concentration–response regression modelling, has not been widely recognised in the genotoxicity mixtures field, and consequently, there is no consensus about their validity and applicability. At one extreme of the spectrum of opinions, it has been claimed that the toxicity of mixtures cannot be predicted from that of its components, e.g. Kocaman and Topaktas (2010), referring to a paper by Marinovich et al. (1996). Conversely, the US National Academy of Sciences, in their 1989 report on toxicants in drinking water (National Research Council 1989), recommended the use of IA for the estimation of risks from mixtures of carcinogens and genotoxic agents, viewing carcinogenesis and genotoxicity as stochastic processes, commensurate with the assumptions underlying IA. Finally, Lutz et al. (2002) found that the joint mutagenic effects (Ames assay) of polycyclic aromatic hydrocarbons could be accurately predicted by CA.

Until recently, studies that allowed comparative evaluations of the validity of CA and IA for predicting combined effects of chemicals with genotoxic modes of action—here understood broadly to include DNA damage, mutations, chromosome damage and induction of micronuclei (MN)—were not available. We previously have begun to address this issue in experiments with seven aneugenic benzimidazole pesticides which induce MN in Chinese hamster ovary (CHO)-K1 cells through a common mechanism (Ermler et al. 2013). As expected in the light of their mechanistic similarity, CA produced accurate predictions of the joint action of these seven benzimidazoles, on the basis of their individual effects, while IA yielded additivity predictions that fell short of the experimentally observed effects. Had we used additivity predictions derived from IA as the basis of assessment, we would have concluded erroneously that the combined effect of benzimidazoles is synergistic.

Here, we present investigations that extend the scope of our earlier mixture studies. We experimentally assessed several mixtures using nine chemicals known to be capable of inducing MN in the cytokinesis-block micronucleus (CBMN) assay in CHO-K1 cells by several different mechanisms (Table 1). We used five aneugenic compounds of which three (vinblastine sulphate, the anthelmintic flubendazole and colchicine) induced MN by blocking microtubule formation through binding to free β-tubulin monomers at the colchicine-binding site. The two remaining aneugens were the anti-fungal drug griseofulvin, which disrupts microtubule polymerisation by binding to a tubulin monomer site different from the colchicine-binding site, and the anti-cancer drug paclitaxel, which induces MN by inhibiting the depolymerisation of microtubules. We also used clastogens: mitomycin C and melphalan (DNA cross-linking chemotherapeutic compounds); doxorubicin hydrochloride (intercalating topoisomerase II inhibitor); etoposide (which inhibits topoisomerase II by binding to DNA grooves).

**Table 1:** Composition of the mixtures tested in the CBMN assay

Compounds	Mechanism of action	Mixture composition and fraction of individual compounds				
		Mixture I (%)	Mixture II (%)	Mixture III (%)	Mixture IV (%)	Mixture V (%)
Aneugens						
Colchicine	Inhibition of microtubule formation (colchicine-binding site)	1.82	–	–	–	–
Flubendazole	Inhibition of microtubule formation (colchicine-binding site)	1.44	34.77	33.12	68.60	94.53
Griseofulvin	Inhibition of microtubule formation (distinct binding site)	96.07	–	–	–	–
Paclitaxel	Inhibition of microtubule depolymerisation (distinct binding site)	0.66	–	15.15	31.40	–
Vinblastine sulphate	Inhibition of microtubule formation (colchicine-binding site)	0.01	–	–	–	–
Clastogens						
Doxorubicin hydrochloride	Topoisomerase II inhibitor (DNA intercalation)	–	2.01	1.92		5.47
Etoposide	Topoisomerase II inhibitor (non-intercalating, binding to enzyme and DNA grooves)	–	8.89	–	–	–
Melphalan	DNA cross-linker (N7 alkylation of guanine)	–	52.29	49.81	–	–
Mitomycin C	DNA cross-linker (sequence specific N alkylations of guanine in 5′-CpG-3′)	–	2.04	–	–	–

Percentages show the fraction of the individual compounds in the mixture

We assessed the applicability of CA and IA to mixtures of chemicals that induce MN through a wide range of different mechanisms.

## Materials and methods

### Chemicals and reagents

Colchicine (CAS 64-86-8), doxorubicin hydrochloride (CAS 25316-40-9), etoposide (4′-demethylepipodophyllotoxin 9-(4,6-O-ethylidene-β-d-glucopyranoside), CAS 33419-42-0), flubendazole [methyl *N*-(6-(4-fluorobenzoyl)-1H-benzimidazole-2-yl)carbamate, CAS 31430-15-6], griseofulvin [(2S)-*trans*-7-chloro-2′,4,6-trimethoxy-6′-methylspiro(benzofuran-2[3H],1′-[2]cyclohexene)-3,4′-dione, CAS 126-07-8], melphalan [4-(bis(2-chloroethyl)amino)-l-phenylalanine, CAS 148-82-3], paclitaxel (CAS 33069-62-4), vinblastine sulphate (CAS 143-67-9) and benzo[α]pyrene (CAS 50-32-8) were purchased from Sigma-Aldrich (Dorset, UK) at the highest purity available. MTT [3-(4,5-dimethylthiazol-2-yl)-2,5-diphenyltetrazolium bromide], acridine orange (AO) and cytochalasin B (10 mg/ml) were also obtained from Sigma. Mitomycin C (CAS 50-07-7) was provided by Calbiochem (Millipore, Watford, UK), paraformaldehyde (PFA) by Avocado chemicals (Lancashire, UK) and dimethyl sulphoxide (DMSO, cell culture grade) and Triton X-100 by VWR (Lutterworth, UK). F12-K cell culture medium and HBSS buffer were purchased from Invitrogen (Paisley, UK).

### Routine cell culture of CHO-K1 cells

The CHO cell line CHO-K1 was purchased from the ATCC (ATCC No CCL-61, LGC standards, Teddington, UK). Cells were routinely grown in 75-cm^2^ canted-neck tissue culture flasks in F-12K medium (Invitrogen) supplemented with 10 % foetal calf serum (FCS; Invitrogen) in a humidified incubator at 37 °C with 5 % CO_2_. Cells were subcultured when confluent over a maximum of 10 passages and were tested routinely for *Mycoplasma* infections.

### CBMN assay

#### Treatment of CHO-K1 cells

The CBMN assay (Fenech 2000) was performed as described earlier (Ermler et al. 2013). Briefly, CHO-K1 cells were seeded in F-12K medium (10 % FCS) at a density of 1.2 × 10^4^ cells/well in 24-well plates and allowed to attach for 24 h before addition of the treatments. All compounds were dissolved in DMSO, and serial dilutions of the chemical or mixture stocks were diluted in F-12K assay medium, the DMSO concentration never exceeding 0.5 %. Eight different concentrations were tested for each chemical or mixture per experiment. Controls were treated in duplicate with solvent (0.5 % DMSO, negative control). Cells were treated for 24 h, and exposure to light was kept to a minimum to avoid UV-induced genotoxicity.

#### Cytokinesis block

Subsequent to treatments, the cells were washed once with F-12K medium, before adding F-12K medium (10 % FCS) supplemented with 3 μg/ml cytochalasin B to block cytokinesis for 18–20 h. After this period, the medium was changed to F-12K medium (10 % FCS) and the cells left to recover for 1–2 h.

#### Slide preparation and staining

The cells were harvested by trypsinisation, counted and centrifuged onto glass slides using a cytocentrifuge for 10 min at 1,200 rpm. The final cell density per slide was kept between 50,000 and 100,000 cells. The cells were immediately fixed in 4 % PFA or 4 % formaldehyde (in PBS) for 10 min at room temperature. The fixed slides were washed for 2 × 5 min in PBS on a shaker, before staining them with 10 μg/ml AO (in ddH_2_O) for 10 min at room temperature. The slides were washed for 2 × 5 min in ddH_2_O on a shaker, then dipped into ddH_2_O, allowed to air-dry and mounted with Vectashield HardSet mounting medium containing DAPI (1.5 μg/ml, Vector Laboratories).

### Automated image acquisition and micronucleus scoring

For automated image acquisition and MN scoring, a Pathfinder™ Cellscan μN platform for automated micronucleus assay scoring (IMSTAR) was used. It was equipped with an Olympus BX41 fluorescence microscope with an automated stage and employed the IMSTAR Pathfinder™ software for image acquisition and analysis. Image acquisition and MN scoring were performed as reported previously (Ermler et al. 2013). Comparison of manual with automated counts revealed that automated counting persistently underestimated MN scores relative to manual counting. This systematic error was consistently observed for different compounds and at different effect concentrations. The underscoring by automated counting was mostly due to the more conservative setting of the scoring algorithm towards avoiding false-positive MN and was comparable to other automated MN scoring systems as discussed in Ermler et al. (2013). Most importantly, automated scoring produced data with sufficiently low inter-experimental variability and high data reproducibility which provided good foundations for mixture experiments. Data output contained the total number of mono- and bi-nucleated cells and the number of mono- and bi-nucleated cells that contained MN. Treatment of cells with aneugens might cause mitotic slippage, i.e. upon prolonged activation of the spindle assembly checkpoint, the cells might escape mitosis and re-enter G1 phase, leading to tetraploid mono-nucleated cells with MN instead of binucleated cells in the CBMN assay (Elhajouji et al. 1998; Hashimoto and Todo 2013). As an exclusive focus on binucleated cells might have led to underestimations of MN frequencies, we also looked at MN induction in mono-nucleated cells. A slightly higher number of MN containing mono-nucleated cells were observed upon treatment with the aneugens in comparison with clastogens. However, with the automated scoring system, it was not possible to distinguish between diploid and the relevant tetraploid cells. Furthermore, the differences between MN frequencies in mono-nucleated cells and those in binucleated cells were only minor and were without impact on the estimated threshold concentrations for the individual mixture components. Our mixture studies were therefore based on MN frequencies in binucleated cells only. For each slide >1,000 binucleated (bn) cells were analysed (unless this was not possible due to cytotoxicity).

### MTT assay for measurement of cytotoxicity

To ensure that cytotoxicity did not have a major impact on MN induction by the chemicals and mixtures, we also determined their cytotoxicity using a modified version of the 3-(4,5-dimethylthiazol-2-yl)-2,5-diphenyltetrazolium bromide (MTT) assay (Mosmann 1983) as described in (Ermler et al. 2013). Briefly, CHO-K1 cells were seeded at 5,000 cells/well in F-12K medium (10 % FCS) in clear plastic 96-well plates. Cells were allowed to attach for 24 h before being treated with the test compounds. Cells were treated similar to the CBMN assay, i.e. for 24 h with test compounds or mixtures, washed and cytokinesis blocked for 18–20 h with cytochalasin B (3 μg/ml) followed by 1 h recovery. All chemicals were dissolved in DMSO and diluted in assay medium, the DMSO concentration never exceeding 0.5 %. Samples were tested in duplicate. Controls were treated with DMSO only (solvent control) or with 1 % Triton X-100 (positive control). Following the treatments, the medium was replaced with MTT-solution (F-12K medium (10 % FCS) containing 250 μg/ml MTT) and incubated for 1 h (reduction of yellow MTT to dark blue formazan crystals by viable cells). After washing the cells with HBSS buffer, the formazan crystals were dissolved in DMSO for 30 min on a shaker. The absorbance was read in a plate reader at 570 and 620 nm. Background correction was performed by subtracting the 620 nm from the 570 nm readings. Data were normalised by subtraction of the average positive control values from the sample values and the average of solvent controls, and then by dividing the corrected sample values by the corrected solvent controls.

### Biostatistical analysis of the CBMN assay

Our methods for the biostatistical analysis of the CBMN assay have been described in detail previously (Ermler et al. 2013) and are briefly outlined below. MN induction in the CBMN assay was measured as the number of binucleated (bn) cells with at least one MN (*N*_MN≥1_) in relation to all binucleated cells (*N*_total_) and expressed as ratio *r*:

1 $$r = \frac{{N_{{{\text{MN}} \ge 1}} }}{{N_{\text{total}} }}.$$

All cells expressed spontaneous levels of MN, which can be observed in untreated control cultures, and these baseline responses were taken into account for regression modelling. Furthermore, for all selected test compounds, we assumed a concentration threshold concept and selected three potential threshold concentration–response models—logit, probit and Weibull—all capable of accurately describing concentration–response data from the CBMN assay. The corresponding functions for a response likelihood *P* at concentration *c* are

2 $${\text{Logit}}\!:\quad P(c) = \left\{ {\begin{array}{*{20}c} {1/\left( {1 + \exp ( - \theta_{1} )} \right)} \hfill &\quad {{\text{for}}\,c \le 1 0^{d} } \hfill \\ {1/\left( {1 + \exp \left( { - \theta_{1} - \theta_{2} \times (\log_{10} (c) - d)} \right)} \right)} \hfill & \quad{{\text{for}}\,c > 1 0^{d} } \hfill \\ \end{array} } \right.$$

3 $${\text{Probit}}\!:\quad P(c) = \left\{ {\begin{array}{*{20}c} {{\text{probnorm}}\,(\theta_{1} )} \hfill &\quad {{\text{for}}\,c \le 10^{d} } \hfill \\ {{\text{probnorm}}\,\left( {\theta_{1} + \theta_{2} \times \left( {\log_{10} (c) - d} \right)} \right)} \hfill &\quad {{\text{for}}\,c > 10^{d} } \hfill \\ \end{array} } \right.$$

4 $${\text{Weibull}}\!:\quad P(c) = \left\{ {\begin{array}{*{20}c} {1 - \exp \left( { - \exp ( - \theta_{1} )} \right)} \hfill &\quad {{\text{for}}\,c \le 1 0^{d} } \hfill \\ {1 - \exp 1\left( { - \exp \left( {\theta_{1} + \theta_{2} \times (\log_{10} (c) - d)} \right)} \right)} \hfill &\quad {{\text{for}}\,c > 1 0^{d} } \hfill \\ \end{array} } \right.$$

where *θ*_1_ and *θ*_2_ are location and scale model parameters, d the threshold model parameter which defines the threshold concentration as *c*_threshold_ = 10^*d*^, and probnorm(*x*) the function that returns the probability that an observation from the standard normal distribution is ≤*x* (inverse of the probit function). The baseline rate of response was defined in the upper part of each conditional equation, i.e. at concentrations below the threshold concentration. All models were fitted separately to each data set, and the best fitting model was selected for each chemical according to a statistical goodness-of-fit criterion (Akaike information). To address the uncertainty of threshold estimations and their consequences on the mixture assessment, we alternatively assumed a non-threshold situation in our data and used the proposed concentration–response models without a threshold model parameter to describe the data. Here the sigmoidal-shaped curve rises from a lower asymptote equalling the baseline (see Scholze et al. 2001 for more details). Only data from concentrations <40 % cytotoxicity (MTT–EC_40_) were included in data analysis. Data analyses were always performed on pooled data sets from at least three independent experiments, a potential extra binomial variation was taken into account by an additional overdispersion parameter, and model parameters were estimated by (restricted) maximum likelihood. All statistical analysis was performed using SAS statistical software version 9.2 (SAS Institute Inc., Cary, NC, USA).

### Mixture predictions

Mixture effects were predicted with the two models for CA and IA that we previously adapted to the use of threshold concentration–response relationships (Ermler et al. 2013). In short, CA is defined for a mixture of n components by

5 $$\sum\limits_{i = 1}^{n} {\frac{{c_{i} }}{{{\text{EC}}x_{i} }} = 1} .$$

In these equations, *c*_*i*_ are the individual concentrations of the substances 1 to n which are present in a mixture that produces the definite effect *x*, and EC*x*_*i*_ denote the equivalent effect concentrations of the single substances, i.e. those concentrations that alone would produce the same quantitative effect *x* as the mixture. The individual concentrations c_i_ sum up to a total concentration *c*_mixture_ that causes the joint effect *E*(*c*_mixture_) = *x*, and thus by definition is the effect concentration ECx_mix_. Equation (5) can be rearranged to

6 $${\text{EC}}x_{\text{mix}} = \left( {\sum\limits_{i = 1}^{n} {\frac{{p_{i} }}{{F_{i}^{ - 1} (x)}}} } \right)^{ - 1}$$

with *p*_*i*_ defined as the prevalence of a mixture component in the mixture, i.e. the ratio of its concentration to the total mixture concentration (*p*_*i*_ = *c*_*i*_/*c*_mixture_), and *F*_*i*_^−1^ the inverse of concentration–response functions from Eqs. (2)–(4), i.e. *F*_*i*_^−1^(*x*) describes the concentration *c* of the *i*th substances that produce an individual effect x, i.e. EC*x*_*i*_ = *F*_*i*_^−1^(*x*). In Ermler et al. (2013), we described in more detail how we deal with varying individual baseline rates from the individual compounds in Eq. (6).

IA can be defined for a mixture of n components by

7 $$E(c_{\text{mixture}} ) = 1 - \prod\limits_{i = 1}^{n} {\left( {1 - E(c_{i} )} \right)}$$

where *E*(*c*_*i*_) denotes the effect caused by the individual compound *c*_1_ of the *i*th compound and *E*(*c*_mixture_) is the total effect of the mixture concentration *c*_mixture_. The individual effects of mixture compounds *E*(*c*_*i*_) are calculated from the concentration–response functions from Eqs. (2)–(4). For concentration–response models with a baseline effect, the single effects have to be corrected first by their individual background baseline estimates (baseline_*i*_), followed by a correction of the total mixture effect by an estimate for the expected baseline for the mixture, i.e.

8 $$E(c_{\text{mix}} ) = 1 + {\text{baseline}}_{\text{mixture}} - \prod\limits_{i = 1}^{n} {\left( {1 - \left( {F_{i} (x) - {\text{baseline}}_{i} } \right)} \right)} .$$

There is no universally accepted procedure for estimating the baseline response for a combination of agents. We used the smallest and highest baseline from all compounds and calculated for each mixture concentration two effect predictions, spanning a range of IA predictions (see Ermler et al. 2013 for more detail).

To extend our assessment to more diverse types of mixtures, when only subsets of components in a mixture were expected to follow the principles of CA, we used a hybrid version of CA and IA. The principle of the hybrid version is as follows: first all compounds presumed to act through a similar mechanism are grouped together and their combined effects predicted according to CA. This approach is not limited to a single group, but in the case of n different mechanisms n different group responses can be predicted. A prediction of the overall effects of the mixture is then derived by using the effects anticipated for these groups, together with the individual effects from all remaining ungrouped compounds as inputs for calculations according to IA. However, the mathematical realisation is less straightforward, mainly as Eq. (6) predicts effect concentrations for CA groups, but not effects (which are required as input in the IA equation). As consequence, an explicit mathematical form describing the total mixture effect as a function of the single substance effects cannot exist, at least for the concentration–response functions from Eq. (2)–(4), and CA mixture effects within each group can only be estimated at a given mixture concentration by numerical methods. Here, we used the bisection method (Burden and Faires 1985), but any other simple root-finding algorithm might be used.

### Mixture experiment design and testing

The mixtures were designed using the concentration–response relationships of the CBMN positive compounds to be included in the respective mixture (Table 2). A fixed mixture ratio approach (Altenburger et al. 2000) with mixture ratios proportional to equi-effective levels was used for all mixtures. To maximise the prediction differences between CA and IA, we chose mixture ratios in proportion to the estimated threshold concentrations of the selected chemicals. The mixture ratios, expressed as fractions of the individual compounds within the different mixtures, are presented in Table 1. Mixture stock solutions at the respective mixture ratios were prepared and serially diluted to cover the effective concentration ranges predicted by CA and IA. In some cases, this meant testing concentrations in the cytotoxic range. The sum of the estimated threshold concentrations was also included in the test concentrations. The effects of all mixtures were assessed experimentally in the CBMN assay in at least three independent experiments and compared to the predictions.

**Table 2:** Model parameters of threshold concentration–response models for all tested single compounds and five mixtures

Compounds	Model	Model parameter			Baseline rate^a^	Threshold concentration	Goodness-of-fit (AIC)	
		$\hat{\theta }_{1}$	$\hat{\theta }_{2}$	$\hat{d}$	(95 % CI) (%)	(95 % CI) [M] (%)	Threshold	No threshold^c^
Aneugens								
Colchicine	Probit	−2.228	1.903	−6.936	1.30 (1.10–1.49)	1.16E−07 (9.50E−08–1.41E−07)	150.9	157.1
Flubendazole^b^	Weibull	−4.358	3.134	−7.035	1.27 (1.14–1.41)	9.22E−08 (7.29E−08–1.16E−07)	160.1	160.8
Griseofulvin	Logit	−4.093	3.735	−5.213	1.64 (1.45–1.83)	6.13E−06 (4.88E−06–7.69E−06)	152.1	154.0
Vinblastine sulphate	Weibull	−3.932	1.216	−9.219	1.94 (1.65–2.23)	6.04E−10 (3.24E−10–1.12E−09)	175.0	172.6
Paclitaxel	Logit	−3.810	2.549	−7.375	2.17 (1.95–2.38)	4.22E−08 (3.37E−08–5.28E−08)	190.5	186.7
Clastogens								
Doxorubicin hydrochloride	Weibull	−3.778	2.756	−8.273	2.26 (2.00–2.52)	5.33E−09 (4.42E−09–6.43E−09)	195.2	195.3
Etoposide	Weibull	−3.872	2.156	−7.628	2.06 (1.83–2.29)	2.36E−08 (1.88E−08–2.96E−08)	191.6	189.8
Melphalan	Weibull	−4.149	2.307	−6.858	1.57 (1.37–1.76)	1.39E−07 (1.16E−07–1.66E−07)	294.4	295.2
Mitomycin C	Probit	−2.027	0.617	−8.268	2.14 (1.84–2.43)	5.40E−09 (3.69E−09–7.88E−09)	166.7	166.4
Mixtures								
I	Weibull	−4.299	2.561	−5.857	1.34 (1.10–1.60)	1.39E−06 (1.05E−06–1.83E−06)		
II	Weibull	−4.088	2.855	−7.018	1.66 (1.45–1.87)	9.59E−08 (8.28E−08–1.11E−07)		
III	Weibull	−4.326	2.577	−7.233	1.31 (1.04–1.57)	5.85E−08 (4.67E−08–7.33E−08)		
IV	Weibull	−4.479	2.036	−7.418	1.13 (0.87–1.39)	3.82E−08 (2.64E−08–5.52E−08)		
V	Weibull	−4.581	2.653	−7.369	1.02 (0.70–1.34)	4.28E−08 (3.01E−08–6.07E−08)		

*AIC* Akaike information criterion (small indicates better fit)

^a^Baseline rate is expressed as percentage; $\hat{\theta }_{1}$, $\hat{\theta }_{2}$ and $\hat{d}$ are estimates of the unknown model parameter *θ*_1_, *θ*_2_ and *d*

^b^Published in Ermler et al. (2013)

^c^Non-threshold model regression fit (Scholze et al. 2001)

## Results

### Concentration–response analysis of individual aneugens and clastogens

**Fig. 1:**
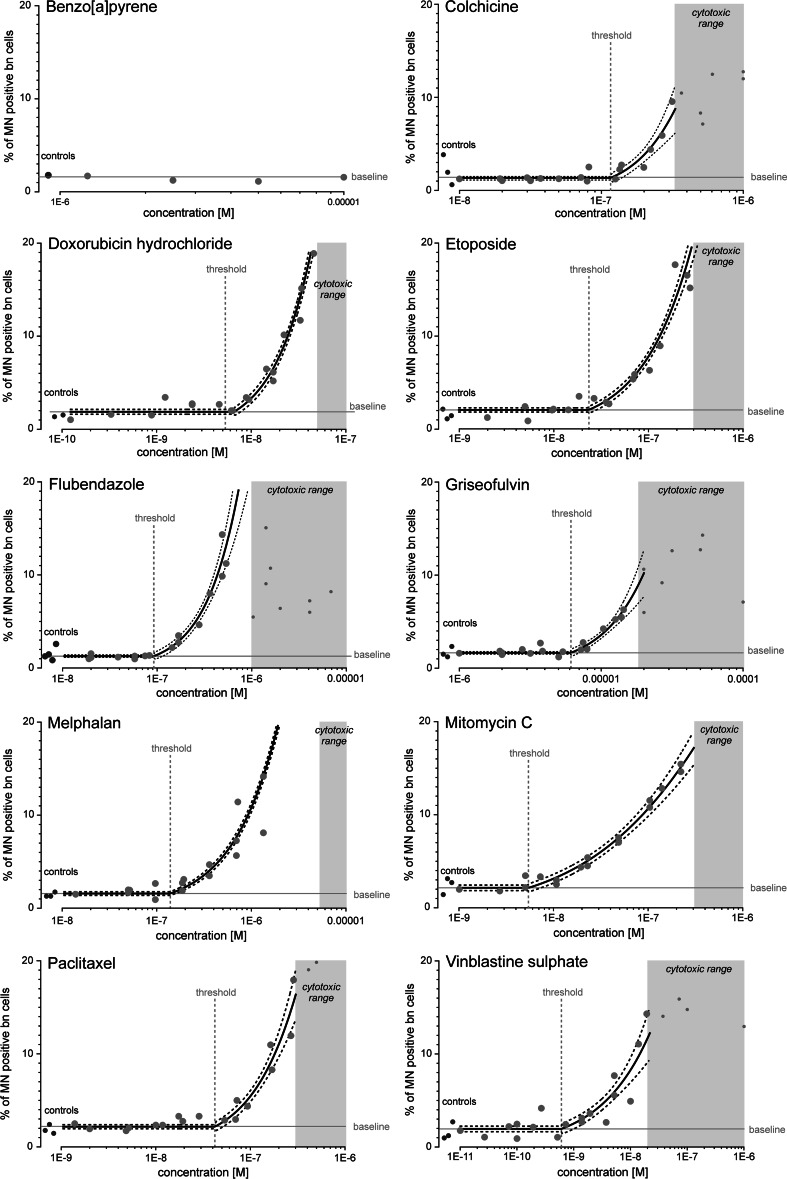
Induction of MN by aneugens and clastogens in the CBMN assay using CHO-K1 cells. MN induction is presented as percentage of MN positive binucleated cells. The *graphs* show the data for at least three independent experiments (*red dots*, exception: benzo[α]pyrene was tested only once); solvent controls are shown on the left (*green dots* as indicated). The regression curves (*thick black lines*) are shown with their 95 % confidence belts (*dashed lines*). Estimated threshold concentrations are indicated by the *vertical dashed lines*. Mean baseline levels of MN within the cells are depicted as *horizontal lines*. The *grey areas* show the cytotoxic concentrations determined in the MTT assay (MTT–EC_40_) (colour figure online)

To provide a basis for predicting and assessing their combined effects, we conducted detailed concentration–response analyses for all individual chemicals included in our mixtures. Each chemical was tested in at least three independent experiments and at eight different concentrations in the CBMN assay. At low concentrations, all chemicals produced MN frequencies not different from those observed in untreated controls, typically between 1.02 and 2.26 % of binucleated cells. As the concentrations increased, MN frequencies did not change until there was a discontinuous rise, indicative of an effect threshold. Above these estimated threshold concentrations, highlighted as vertical dashed lines in Fig. 1, the compounds induced MN in a concentration-dependent manner, in a nonlinear fashion. The exception to this was benzo[a]pyrene, which proved ineffective in these studies, presumably because the levels of cytochrome P450 isoforms required to convert benzo[a]pyrene into active epoxides were too low in CHO-K1 cells. To describe these concentration–response relationships, we employed nonlinear regression models which included a threshold parameter. These nonlinear models (listed in Table 2, together with model parameters, including estimated thresholds) generally described the data better than the widely used hockey stick models with their linear functions at concentrations above thresholds (Lutz and Lutz 2009).

Of the tested aneugens, vinblastine sulphate was the most potent, with an estimated threshold concentration of 0.6 nM, followed by paclitaxel (42 nM), flubendazole (92 nM), colchicine (116 nM) and griseofulvin, which was the least potent at 6.13 μM. The most potent clastogen was doxorubicin hydrochloride with an estimated threshold of 5.33 nM, closely followed by mitomycin C (5.4 nM), etoposide (23.6 nM) and melphalan (139 nM) (Table 2).

As with benzimidazoles (Ermler et al. 2013), cytotoxicity led to considerably increased variability in MN frequencies and a downturn in effect, which was quite pronounced in the case of colchicine, griseofulvin and vinblastine (Fig. 1). Clearly, cytotoxicity was a confounding factor that complicated concentration–response analysis. It therefore became necessary to establish concentration ranges associated with cytotoxicity, with the aim of excluding data points confounded by cytotoxicity from concentration–response analyses. We considered the cytokinesis-block proliferation index (CBPI) as a diagnostic criterion, but found previously that this method produced unreliable results especially with aneugens (Ermler et al. 2013). We therefore tested all compounds in the MTT assay (Table 3) and used effect concentrations associated with 40 % cytotoxicity (MTT–EC_40_) as a cut-off criterion above which data from the CBMN assay were omitted from concentration–response analysis for both aneugens and clastogens. We chose the MTT–EC_40_ instead of up to 55 ± 5 % cytotoxicity as suggested by OECD guideline 486 (2010) because of a considerable increase in inter-experimental data variability above 40 % cytotoxicity, which would have impacted negatively on regression modelling. The grey-shaded areas in the graphs in Fig. 1 show the concentration ranges above the MTT–EC_40_. With all aneugens, concentrations associated with cytotoxicity did overlap with those causing MN, but this was not the case with the clastogens we tested.

**Table 3:** Cytotoxicity of test compounds (MTT assay)

Substances	Concentration–response function						EC_10_	EC_20_	EC_40_
	RM	$\hat{\theta }_{1}$	$\hat{\theta }_{2}$	$\hat{\theta }_{3}$	$\hat{\theta }_{\hbox{min} }$	$\theta_{\hbox{max} }$	M [CI]	M [CI]	M [CI]
Aneugens									
Colchicine	Logit	−40.12	−5.70	–	0.56	1	5.56E−8 [4.10E−8–7.55E−8]	8.47E−8 [6.78E−8–1.06E−7]	2.32E−7 [1.41E−7–3.84E−7]
Flubendazole^a^	Logit	−37.62	−5.59	–	0.57	1	1.15E−7 [8.03E−8–1.64E−7]	1.77E−7 [1.38E−7–2.28E−7]	5.44E−7 [3.03E−7–9.77E−7]
Griseofulvin	Logit	−28.44	−5.71	–	0.50	1	5.94E−6 [2.81E−6–1.25E−5]	8.83E−6 [5.22E−6–1.49E−5]	1.83E−5 [8.65E−6–3.88E−5]
Paclitaxel	Logit	−32.66	−4.72	–	0.50	1	6.20E−8 [3.46E−8–1.11E−7]	1.00E−7 [6.84E−8–1.47E−7]	2.42E−7 [1.43E−7–4.11E−7]
Vinblastine sulphate	Logit	−42.93	5.11	–	0.54	1	2.24E−9 [1.41E−9–3.58E−9]	3.55E−9 [2.54E−9–4.96E−9]	9.40E−9 [5.55E−9–1.59E−8]
Clastogens									
Doxorubicin hydrochloride	Logit	8.59	−1.30	–	−483	1	1.01E−7 [5.79E−8–1.78E−7]	3.46E−7 [2.41E−7–4.96E−7]	1.18E−6 [9.78E−7–1.42E−6]
Etoposide	Weibull	−2.58	−0.52	–	0*	1	2.32E−7 [5.76E−8–9.37E−7]	1.15E−6 [4.66E−7–2.86E−6]	1.44E−5 [2.77E−6–7.43E−5]
Melphalan	Weibull	−11.73	−2.32	–	0*	1	3.88E−6 [2.46E−6–6.12E−6]	5.54E−6 [3.58E−6–8.56E−6]	>5.2E−6
Mitomycin C	Logit	−4.56	−1.69	–	45.22	1	4.53E−7 [2.29E−7–8.96E−7]	1.17E−6 [7.52E−7–1.82E−6]	3.03E−6 [2.38E−6–3.87E−6]
Mixtures									
I	Weibull	−16.35	−2.92	–	0.59	1	1.95E−6 [1.60E−6–2.38E−6]	3.35E−6 [2.85E−6–3.95E−6]	>1.00E−5 n.d.
II	Logit	−36.13	−6.12	–	0*	1	5.49E−7 [4.72E−7–6.38E−7]	7.45E−7 [6.80E−7–8.16E−7]	1.08E−6 [9.74E−7–1.19E−6]
III	Logit	−57.68	−8.87	–	0.59	1	2.35E−7 [1.78E−7–3.10E−7]	3.11E−7 [2.54E−7–3.82E−7]	7.77E−7 [7.40E−8–8.16E−6]
VI	Logit	−55.66	−8.19	–	0.58	1	1.16E−7 [9.46E−8–1.43E−7]	1.57E−7 [1.37E−7–1.79E−7]	3.68E−7 [2.25E−7–6.02E−7]
V	Logit	−59.95	−8.99	–	0.48	1	1.49E−7 [1.08E−7–2.05E−7]	1.91E−7 [1.52E−7–2.40E−7]	2.95E−7 [2.18E−7–3.98E−7]

EC20, EC10: concentration provoking 20 and 10 % lower OD readings to the negative controls, respectively. Values in brackets denote the upper and lower limits of the approximate 95 % confidence interval; the column “RM” indicates the mathematical regression function as defined at Scholze et al. (2001): $\hat{\theta }_{1} ,\hat{\theta }_{2} ,\hat{\theta }_{3} ,\hat{\theta }_{\hbox{min} }$ estimated model parameters, given for concentrations expressed in M (rounded values), $\theta_{\hbox{max} }$were not estimated, but set to 1 relating to the mean value of the negative controls; * hold fixed; “>” indicates highest test concentration

^a^Published in Ermler et al. (2013)

### Prediction and assessment of combination effects

#### Mixture of five aneugens with differing sites of action

**Fig. 2:**
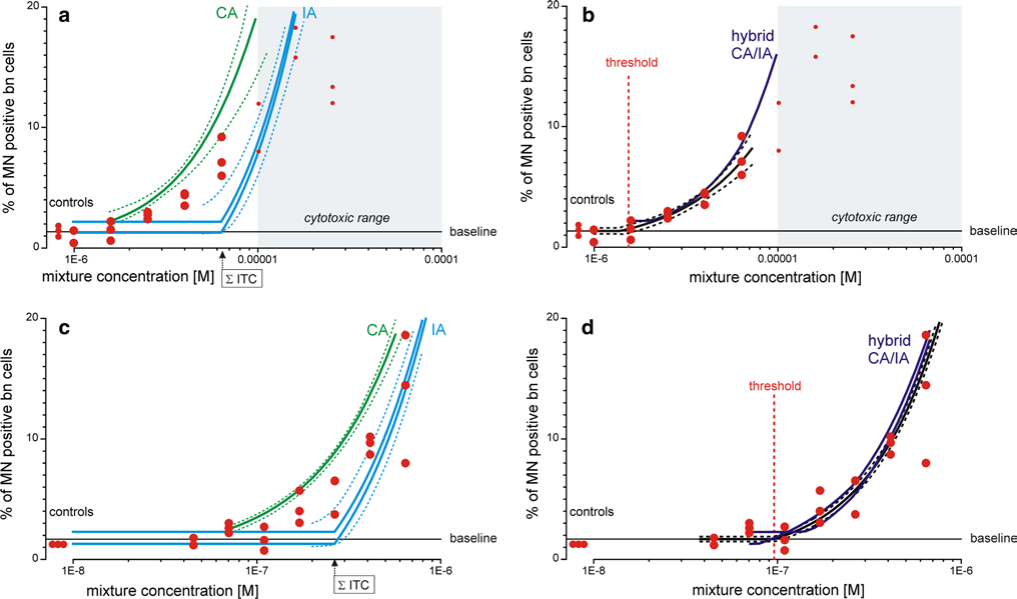
Predicted and observed induction of MN by two mixtures of aneugens or aneugens and clastogens in the CBMN assay. Mixture I was composed of flubendazole, colchicine, griseofulvin, paclitaxel and vinblastine (**a**), and mixture II of flubendazole, doxorubicin, etoposide, melphalan and mitomycin C (**c**). Prediction curves were derived from CA (*green curves* in **a**, **c** as labelled) and IA (*light blue* curves in **a**, **c** as labelled), with *dashed lines* as the respective 95 % confidence belts. Prediction curves were also generated from a hybrid CA/IA model (*dark blue* lines in **b** and **d** as labelled) for mixture I (**b**) and mixture II (**d**), with compounds grouped according to strict criteria of similar and dissimilar mechanism of action. All mixtures were designed at a ratio of the estimated threshold concentrations of the individual compounds and tested as dilution series (the mixture concentrations corresponding to the sum of the individual threshold concentrations are indicated as ΣITC). Data are shown from at least three independent experiments (*red dots* **a**–**d**), together with their regression curves (*thick black lines*) and 95 % confidence belts (*dashed lines* **b**, **d**). Threshold concentrations (*vertical dashed lines*) and mean baseline levels of MN (*horizontal line*) were estimated by regression analysis (see Table 2 for more information). The *grey areas* show the cytotoxic concentrations determined in the MTT assay (MTT–EC_40_) (colour figure online)

We composed a mixture (Mixture I) of inhibitors of microtubule polymerisation colchicine, flubendazole, vinblastine sulphate and griseofulvin, and paclitaxel which inhibits microtubule depolymerisation. The mixture ratio was determined using the chemicals’ estimated threshold concentrations (Table 1). The regression models constructed for the single chemicals were used to calculate mixture effects predictions according to CA and IA. The resulting prediction curves were discriminating, with approximately fourfold higher threshold concentrations predicted by IA (Fig. 2a). Due to a degree of between-experiment variability in the background MN frequencies, two IA curves had to be calculated, one based on the lowest and the other on the highest observed baseline. CA predictions are less sensitive to these baseline variations (Ermler et al. 2013).

The observed MN frequencies of Mixture I fell between the extremes of the prediction window defined by CA and IA (Fig. 2a; Table 4), with neither CA nor IA providing good approximations of the experimental data. When all five chemicals were combined at their individual threshold concentrations, a MN frequency of approximately 7 % was measured, which was well above the baseline levels predicted by IA for this combination.

**Table 4:** Statistical uncertainty of predicted and observed effect concentrations for mixtures

MN induction	Effect concentration ECx_mix_ [M]					
	Observed		Predicted by CA		Predicted by IA	
	Mean	95 % CI	Mean	95 % CI	Mean^a^	95 % CI
Mixture I: five aneugens (ratio as defined in Table 1)						
5 %	4.59E−6	[4.17E−6–5.06E−6]	**3**.**24E**−**6**	[3.00E−6–3.54E−6]	**8**.**07E**−**6**–**8**.**53E**−**6**	[6.50E−6–9.36E−6]
10 %	8.77E−6	[7.27E−6–1.06E−5]	**5**.**72E**−**6**	[5.31E−6–6.88E−6]	1.08E−5–1.13E−5	[9.70E−6–1.24E−5]
Mixture II: flubendazole and four clastogens (ratio as defined in Table 1)						
5 %	2.36E−7	[2.22E−7–2.51E−7]	**1**.**47E**−**7**	[1.36E−7–1.56E−7]	**3**.**44E**−**7**–**3**.**74E**−**7**	[2.88E−7–4.15E−7]
10 %	4.22E−7	[4.06E−7–4.37E−7]	**3**.**01E**−**7**	[2.90E−7–3.15E−7]	**4**.**92E**−**7**–**5**.**23E**−**7**	[4.38E−7–5.68E−7]
Mixture III: four dissimilarly acting compounds (ratio as defined in Table 1)						
5 %	2.02E−7	[1.94E−7–2.10E−7]	**1**.**70E**−**7**	[1.56E−7–1.80E−7]	**3**.**68E**−**7**–**4**.**03E**−**7**	[3.11E−7–4.37E−7]
10 %	3.94E−7	[3.83E−7–4.07E−7]	**3**.**18E**−**7**	[3.06E−7–3.38E−7]	**5**.**32E**−**7**–**5**.**66E**−**7**	[4.79E−7–6.04E−7]
Mixture IV: flubendazole and paclitaxel (ratio as defined in Table 1)						
5 %	2.10E−7	[1.93E−7–2.30E−7]	**1**.**64E**−**7**	[1.41E−7–1.79E−7]	2.23E−7–2.52E−7	[1.80E−7–2.84E−7]
10 %	4.75E−7	[3.99E−7–5.64E−7]	**3**.**02E**−**7**	[2.81E−7–3.39E−7]	3.73E−7–3.98E−7	[3.39E−7–4.33E−7]
Mixture V: doxorubicin hydrochloride and flubendazole (ratio as defined in Table 1)						
5 %	1.73E−7	[1.60E−7–1.88E−7]	**1**.**12E**−**7**	[1.04E−7–1.22E−7]	1.55E−7–1.75E−7	[1.37E−7–1.94E−7]
10 %	3.24E−7	[2.81E−7–3.73E−7]	**1**.**98E**−**7**	[1.87E−7–2.14E−7]	2.51E−7–2.71E−7	[2.34E−7–2.89E−7]

*CA* concentration addition, *IA* independent action, *CI* confidence interval

^a^Prediction ranges are calculated assuming lowest or highest observed baseline; significance between predicted and observed EC_*X*_ values was judged as a non-overlapping of their 95 % percentile bootstrap CIs and is shown in bold

Next, we examined whether the use of a hybrid CA/IA prediction concept would provide better descriptions of the experimental data. To this end, we first grouped the inhibitors of microtubule polymerisation colchicine, flubendazole, griseofulvin and vinblastine sulphate together and predicted their effects by CA. A prediction of the overall effects of the mixture was derived by using the effects anticipated for this group, together with the concentration–response relationship for paclitaxel as inputs for calculations according to IA. This yielded a combination effect prediction that matched the experimental data very well (Fig. 2b).

The grey-shaded areas show the concentrations above which cytotoxicity caused increased data variability, and data in this range were excluded from regression analysis.

#### Mixture of an aneugen and four clastogens

In an effort to compose a mixture which could be expected to match more closely the principles of IA, we applied stricter criteria in terms of varying mechanisms of action and chose the aneugen flubendazole together with the clastogens doxorubicin hydrochloride, etoposide (both topoisomerase II inhibitors, but by different mechanisms), melphalan and mitomycin C (both DNA cross-linking agents but by differing mechanisms), Mixture II (Table 1). Again, CA and IA produced quite different prediction curves (Fig. 2c). By taking account of the variability between baseline levels from different experiments, we derived two curves for IA, which were located closely together. Still, the observed combination effects of these five agents were larger than those anticipated by IA, but fell short of those calculated according to CA. Again, this mismatch prompted us to assess whether a hybrid prediction model would yield better approximations of the observed effects. Accordingly, we combined the two topoisomerase II inhibitors, doxorubicin hydrochloride and etoposide, in one group and the alkylating agents, melphalan and mitomycin C, in a second group and calculated the corresponding group effects separately by using CA. The resulting two CA predictions were then combined with the concentration–response data for flubendazole to derive overall predictions according to IA. As before, we had to accommodate the variability in the baseline levels and obtained two quite closely matched prediction curves. These predictions agreed very well with the observed effects of the combination (Fig. 2d).

#### Mixture of two aneugens and two clastogens

**Fig. 3:**
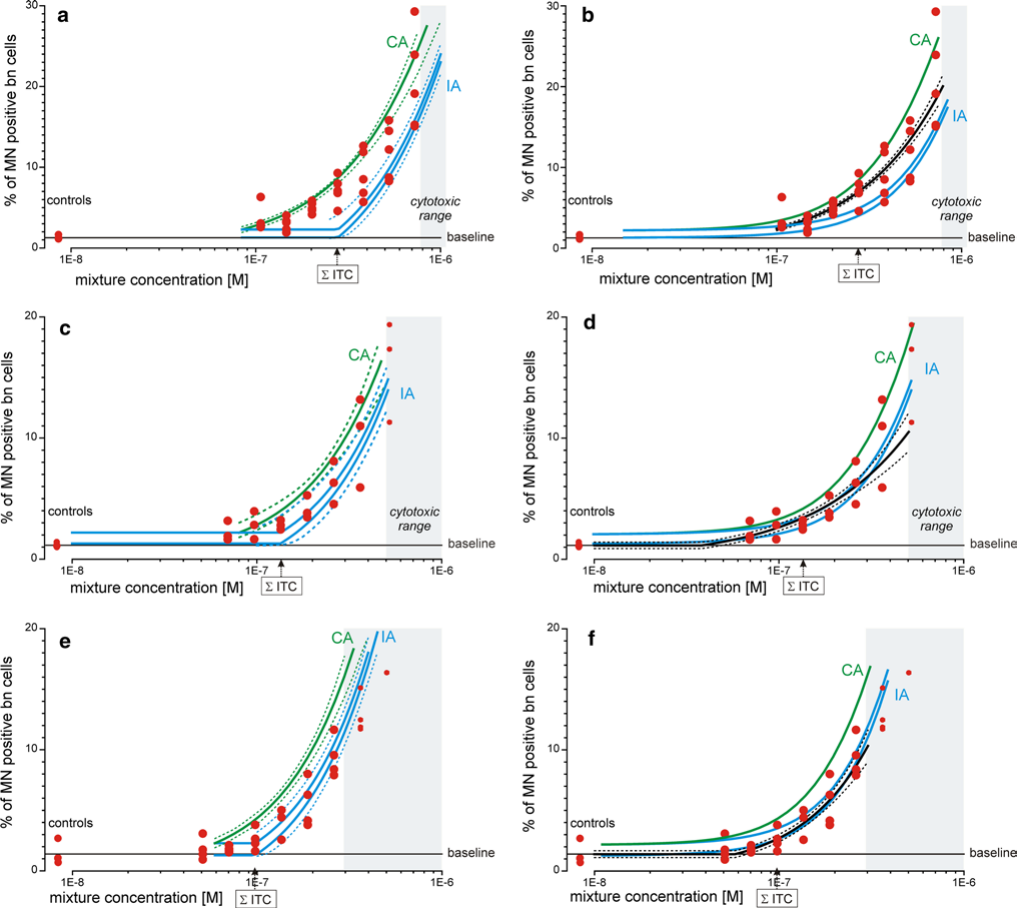
Predicted and observed induction of MN by three mixtures of aneugens or aneugens and clastogens in the CBMN assay. Mixture III was composed of flubendazole, paclitaxel, doxorubicin and melphalan (**a**), mixture IV of flubendazole and paclitaxel (**c**), and mixture V of flubendazole and doxorubicin (**c**). Prediction curves were derived from CA (*green curves* as labelled) and IA (*light blue curves* as labelled), with *dashed lines* the respective 95 % confidence belts. Prediction curves were re-calculated by using only non-threshold regression models and are shown for mixture III (**b**), mixture IV (**d**) and mixture V (**f**). All mixtures were designed at a ratio of the estimated threshold concentrations of the individual compounds and tested as dilution series (the mixture concentration corresponding to the sum of the individual threshold concentrations is indicated as ΣITC). Data shown are from at least three independent experiments (*red dots*), together with their regression curves and 95 % confidence belts (*thick black curves* with *dashed lines*, **b**, **d** and **f**). Mean baseline levels of MN (*horizontal line*) were estimated by regression analysis (see Table 2 for more information). The *grey areas* show the cytotoxic concentrations determined in the MTT assay (MTT–EC_40_) (colour figure online)

In attempting to define a reference case for IA, in Mixture III, we combined two aneugens with diametrically opposed mechanisms of action, flubendazole and paclitaxel, with the clastogens doxorubicin (topoisomerase II inhibitor) and melphalan (alkylating agent) (Table 1). On the basis of the threshold-dependent regression models for these four chemicals (Fig. 1; Table 2), we obtained prediction curves of combination effects according to CA and IA that were separated by a factor of 2–3 on the concentration axis (Fig. 3a). The experimental data showed relatively high between-experiment variability. The observed combination effects agreed better with CA at low mixture concentrations, up to the total mixture concentration equivalent to the sum of the individual estimated thresholds of all single components. At this point, IA predicted MN frequencies similar to background levels, but the observed responses were significantly higher. Beyond that concentration, the observed MN frequencies fell within the window defined by the two predictions.

IA predictions in the range of small effects are strongly affected by the quality of the regression models for the single components in the corresponding concentration ranges. We therefore assessed whether the concentration–response relationships for the individual compounds were described better by regression models without a threshold term and whether the threshold parameters included in our original regressions might have produced a downward bias of the predicted IA effects. We therefore calculated CA and IA predictions using regression models for all single components that did not include a threshold parameter. As expected, this shifted the IA predicted effects at low mixture predictions slightly upwards, towards the experimentally observed values (Fig. 3b), but without substantially improving the agreement between prediction and observation (Table 2).

#### Binary mixtures

Finally, we tested whether the effects of binary combinations of agents with strictly different mechanisms of actions could be approximated by IA. Accordingly, we predicted and assessed the effects of a mixture of flubendazole and paclitaxel, Mixture IV, and of flubendazole and doxorubicin, Mixture V (Table 1). For both combinations, we obtained CA and IA prediction curves located relatively closely together, with sometimes overlapping 95 % confidence belts (Fig. 3c, e). In both cases, the experimental data came quite close to the effects predicted by IA. We also calculated the IA predictions on the basis of threshold-independent regression models (Fig. 3d, f), and this produced a prediction curve in better agreement with the observed MN frequencies. CA led to overestimations of the experimentally observed combination effects.

## Discussion

We have previously shown that chemicals capable of inducing MN by strictly similar mechanisms (disruption of microtubule polymerisation) act together according to the principles of CA (Ermler et al. 2013). In the present study, we relaxed the similarity criteria by which we selected our mixture components and investigated the joint effects of chemicals that produce MN through a variety of different mechanisms. Our aim was to design mixture experiments that were decisive in determining whether the principles of CA were fulfilled or whether IA was applicable. However, despite their differing conceptual origins, CA and IA frequently produce very similar predictions of the combined effects of the same mixture. The factors that drive the prediction differences between CA and IA are well understood (Drescher and Boedeker 1995) and include mixture ratio, steepness of the concentration–response curves of the individual mixture components, the effect magnitude considered for analysis and the number of components included in the mixture. To achieve our aims, we therefore maximised the number of mixture components, as far as possible. More importantly, we chose mixture ratios in proportion to the estimated threshold concentrations of the selected chemicals. This had the added advantage of offering the opportunity to test a central tenet of the IA concept: if the principles of IA apply, mixture effects are not expected to rise above background MN frequencies if all chemicals are present at their respective threshold concentrations.

None of the mixtures investigated here produced combination effects described well by CA. Instead, we exposed an assessment dilemma for two of our mixtures, Mixtures I and II, where the observed MN frequencies fell between the boundaries defined by the CA and IA predictions. Since CA predicted higher combination effects than IA in both cases, the observed responses can be evaluated as antagonisms in relation to CA, or as synergisms relative to IA. We were able to resolve this unsatisfactory situation by application of hybrid CA/IA models, where chemicals were first grouped according to criteria of similarity, their joint effects predicted by CA and finally the overall mixture effects anticipated by using IA. This procedure yielded mixture effect predictions in good agreement with the experimental observations.

We anticipated that the four-component mixture (Mixture III) composed of flubendazole, paclitaxel, doxorubicin and melphalan should follow IA since these agents are known to produce MN by a variety of mechanisms. However, the observed responses were better approximated by CA, up to concentrations equivalent to the sum of the estimated thresholds for all single components. Beyond that concentration, neither concept provided satisfactory approximations of the empirical MN frequencies. It seems, therefore, that the fundamental principles of IA, the statistical concept of independent random events, cannot be translated fully to our biological test system with multi-component mixtures. This might be due to overlapping stress and repair responses and other signalling pathways in response to the treatments, which might violate the principles of IA, but ultimately, we are unable to advance convincing explanations at this stage. The application of CA/IA hybrid models to this mixture was not feasible, as an assignment of components to groups according to similarity criteria was not possible, due to their distinct mechanisms.

We venture that the poor agreement between IA and the observed MN frequencies with Mixture III might be related to biased IA predictions resulting from inaccuracies in estimating threshold concentrations for the single compounds. The accuracy of IA predictions depends strongly on reliable estimations of small effects associated with low concentrations. Inaccuracies with a bias in the same direction can sum up to quite large errors, especially with larger numbers of components. Mixture effect predictions derived from CA are far more robust in this respect. The variations that inevitably occur in every data set determine a statistical detection limit below which the reliable estimation of effects is no longer possible. This means that the functional form of empirical regression models in the range of effects below this statistical detection limit can neither be rejected nor confirmed empirically. The existence (or otherwise) of an effect threshold for MN can therefore only be speculated upon, but not be determined by measurement (Slob 1999). In this situation, the only avenue open to us to support the choice between a regression model with a threshold model parameter and its threshold-independent version was to use global goodness-of-fit criteria. In using such criteria, we did not encounter an example where the inclusion of a threshold parameter for any of our single chemical data sets led to significantly poorer goodness-of-fit (Table 2). In most cases, threshold-based regression models even provided slightly better data descriptions. However, it should be emphasised that goodness-of-fit judgements always have to be based on the entire range of data and cannot be restricted to the range of low effects. Accordingly, we derived estimated threshold concentrations from a statistical model that described all the data for a single chemical, and not only those in the low concentration range. For this reason, a threshold represents an estimate of means. As a result, it may happen that certain responses around the threshold estimate are located above the mean estimate, but still within the 95 % confidence belt of the mean. However, such responses will always be larger than the threshold estimate of the regression model. Since the regression models form the basis of the IA prediction, combination effects at threshold concentrations can only be underestimated by IA, but never overestimated and this may well have introduced a downward bias. Within the confines of the IA concept, that bias could only be removed by using threshold-independent regression models for the single mixture components, but at the price of an inferior goodness-of-fit. This price was difficult to justify, considering that the resulting upward shift in the IA predicted mixture effects still did not describe the data well (Fig. 3b). The uncertainties associated with estimating low-level effects also precluded us from arriving at firm conclusions regarding one hallmark of the IA concept, namely that combination effects are not expected when all mixture components are combined at zero effect levels, here understood as background MN frequencies.

For the two binary mixtures (Mixtures IV and V), composed with the intention of realising the principles of dissimilar action, IA did prove to be a reasonable approach for approximating the experimentally observed effects. Other studies have demonstrated that MN induced by binary mixtures of two methylating agents (similar action) could be predicted by CA, whereas binary mixtures with methylating agents and topoisomerase inhibitors (dissimilar action) led to effects that fell between the CA and IA prediction in one case and were smaller than anticipated by IA in another case (Lutz et al. 2005). The only reference cases for IA for multi-component mixtures were established in experiments with strictly dissimilarly acting mixtures in bacteria and algae (Backhaus et al. 2000; Faust et al. 2003). This is significant, because the applicability of IA to mammalian systems has been questioned on grounds of principle (Berenbaum 1989), and our findings with the four compound mixture appear consistent with this.

In practice, situations where IA will be the correct prediction approach are not likely to be encountered frequently. The number of chemicals capable of inducing MN by far exceeds the number of different mechanisms available for MN formation. When applied to realistic exposure scenarios, this means that it is highly likely that several chemicals will exhibit similar mechanisms and produce combined effects according to CA. To aggregate different classes of mechanisms, the use of hybrid CA/IA models would be called for. In such cases, the application of IA will lead to an underestimation of the joint effect (see Mixtures I and II, and Ermler et al. 2013). The degree of underestimation will depend, ceteris paribus, largely on the number of mixture components.

The use of hybrid CA/IA models for the assessment of experimental data may not always be straightforward. It requires clear criteria for the grouping of chemicals according to similar mechanisms. For chemicals capable of inducing MN, a classification into aneugens and clastogens suggests itself as a starting point, but additional information will be required to arrive at finer groupings. For clastogens, a consideration of types of DNA damage (cross-links, intercalation, etc.) might prove productive, but more experience with a wider range of genotoxicants capable of inducing MN will be necessary to draw firm conclusions. It would also need to be taken into account that some compounds exhibit more than one mechanism of action.

While these considerations will be useful when it comes to the evaluation of experimental data, we have doubts whether they will be relevant for the assessment of combination effects of aneugens and clastogens in risk assessment practice. The application of IA, or of hybrid CA/IA models, requires detailed concentration–response data in the range of low effects, which is rarely available. Much less demanding are the data requirements for using CA [reviewed by Kortenkamp et al. (2009)], and CA has the advantage of yielding conservative mixture effect predictions.

In considering whether our observations with the in vitro CBMN assay have relevance to other cell-based systems, or indeed to in vivo MN models, it will be necessary to consider that the CHO-K1 cells employed in our experiments harbour a mutated *TP53* gene. The use of p53 compromised cells has been questioned recently, with the argument that a dysfunctional p53 might lead to the induction of MN in cells that would otherwise have undergone repair or apoptosis (Fowler et al. 2012; Kirkland et al. 2007). This line of argument is no doubt relevant in the context of discussions about extrapolations in a risk assessment and regulatory context, but does not invalidate our observations. It is conceivable that differences in p53 status have an impact on differences in the sensitivity of cells to MN-inducing agents, although evidence from experiments with p53 deficient variants of human TK6 cells have revealed little influence of p53 status on MN formation (Hashimoto et al. 2011; Honma and Hayashi 2011). Furthermore, deviations from additivity for the combined effects of gamma irradiation and ethyl methanesulfonate seemed to be species specific rather than dependent on p53 status (Lutz et al. 2002). In any case, functional p53 is not likely to lead to complete protection against formation of MN, although the potency of chemicals in inducing MN might be affected. However, there is no reason to suspect that alterations in the cells’ reaction to the effects of individual mixture components will change the general principles which govern their joint action. We therefore expect that our findings have relevance to MN formation in other cell-based systems and to in vivo MN models.

It remains to be seen whether the principles of joint action that we established for MN-inducing chemicals are also applicable to other genotoxicity endpoints, such as gene mutations, chromosome mutations or carcinogenicity. The experiences with a mixture of benzo[a]pyrene, benz[a]anthracene and dibenz[a, c]anthracene in a bacterial system (Ames assay) communicated by Lutz et al. (2002) show that CA provided good approximations of the observed joint effects, which was explained in terms of the similarity of the mode of mutagenic action of the tested chemicals, i.e. formation of a similar type of DNA adducts.

In conclusion, our study has established basic principles of joint action of chemicals that affect an endpoint of relevance to genotoxicity. We demonstrate that it is possible to approximate, often fairly accurately, the combined effects of MN-inducing chemicals when their single effects are known. Our study also exposes the need to re-examine the numerous claims of synergisms that have appeared in the specialist literature. These claims stem from an experimental approach based on comparisons of the effects of the mixture with those of the most toxic component, where synergisms are declared when the mixture effect is larger than the effect of the most potent component. It has been frequently overlooked that these observations may also be compatible with additive mixture effects. Proper re-evaluations of the published data in terms of compatibility with ideas about additive effects are not possible in most cases, because concentration–response data of the single mixture components were not provided [see the review by Kortenkamp et al. (2009)]. It is to be hoped that the study of genotoxic mixture effects will be enriched in the future by embracing the theory development that has taken place in other areas of mixture toxicology during the last two decades.
